# Minority and Majority Charge Carrier Mobility in Cu_2_ZnSnSe_4_ revealed by Terahertz Spectroscopy

**DOI:** 10.1038/s41598-018-32695-6

**Published:** 2018-09-27

**Authors:** Hannes Hempel, Charles J. Hages, Rainer Eichberger, Ingrid Repins, Thomas Unold

**Affiliations:** 1 Department Structure and Dynamics of Energy Materials, Helmholtz-Zentrum Berlin für Materialien und Energie GmbH, Hahn-Meitner-Platz 1, 14109 Berlin, Germany; 2 Institute for Solar Fuels, Helmholtz-Zentrum Berlin für Materialien und Energie GmbH, Hahn-Meitner-Platz 1, 14109 Berlin, Germany; 3 National Renewable Energy Laboratory, 15013 Denver West Parkway, Golden, CO 80401-3305 USA

## Abstract

The mobilities of electrons and holes determine the applicability of any semiconductor, but their individual measurement remains a major challenge. Here, we show that time-resolved terahertz spectroscopy (TRTS) can distinguish the mobilities of minority and majority charge carriers independently of the doping-type and without electrical contacts. To this end, we combine the well-established determination of the sum of electron and hole mobilities from photo-induced THz absorption spectra with mobility-dependent ambipolar modeling of TRTS transients. The method is demonstrated on a polycrystalline Cu_2_ZnSnSe_4_ thin film and reveals a minority (electron) mobility of 128 cm^2^/V-s and a majority (hole) carrier mobility of 7 cm^2^/V-s in the vertical transport direction relevant for light emitting, photovoltaic and solar water splitting devices. Additionally, the TRTS analysis yields an effective bulk carrier lifetime of 4.4 ns, a surface recombination velocity of 6 * 10^4^ cm/s and a doping concentration of ca. 10^16^ cm^−3^, thus offering the potential for contactless screen novel optoelectronic materials.

## Introduction

**Figure 1:**
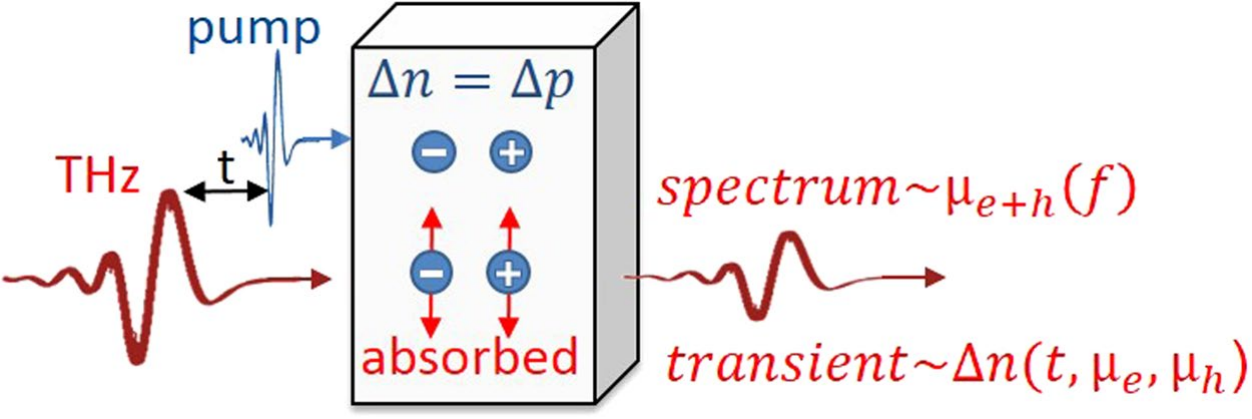
Principle of mobility derivation by time resolved THz spectroscopy (TRTS). Traditionally, TRTS derives the added mobility of electrons and holes *µ*_*e*+*h*_ from the spectrum of the pump-induced THz absorption. We distinguish the mobilities of electrons *µ*_*e*_ and holes *µ*_*h*_ by modeling TRTS transients.

TRTS is a widely used pump-probe technique to measure the dispersive AC-mobility *µ*_*e*+*h*_(*f*) of photo-excited charge carriers at THz frequencies, as well as the decay of the photoconductivity from femtoseconds to nanoseconds shown in Fig. 1 ^1–3^. The outstanding benefits of this technique include its non-destructive and contactless nature as well as high time resolutions of <100 fs. Recently TRTS was extended to be used in reflection mode^4^ and to derive mobilities for thin films on highly conductive substrates^5^.

A major advantage, and at the same time a potential disadvantage, of TRTS is the measurement sensitivity to all pump-induced charge carriers, i.e. both electrons and holes. This makes both types of carriers accessible – but indistinguishable. Accordingly, the added electron and hole mobility *µ*_*e*+*h*_ is generally derived from the photo-induced THz absorption spectra as shown in Fig. 1. In this work we address this issue by analysing TRTS transients and derive both the electron and hole mobility on the same Cu_2_ZnSnSe_4_ thin film sample.

During the last years Cu_2_ZnSnSe_4_ has evolved as a promising compound semiconductor material for solar cells. It is structurally closely related to the more established Cu(In,Ga)Se_2_ chalcopyrite absorber material, but it has the advantage to contain only earth-abundant elements. As the device efficiency of Cu_2_ZnSnSe_4_ solar cells still lags significantly behind record efficiencies of chalcopyrite or CdTe solar cells, fundamental studies of the transport and recombination behavior are expected to identify current performance bottle-necks.

First, we demonstrate the determination of the sum of electron and hole mobility of the Cu_2_ZnSnSe_4_ thin film from TRTS spectra, illustrating the state-of-the-art of TRTS sum mobility measurements. Next, the transient model is developed including dominant first order bulk and surface recombination deduced from wavelength and injection dependent TRTS transients, and ambipolar diffusion verified experimentally by THz emission spectroscopy. Based on these dynamics - ambipolar diffusion, surface, and bulk recombination - TRTS transients are modeled with the continuity equation and the ambipolar diffusion coefficient is determined. Finally, the ambipolar diffusion coefficient and the mobility sum are combined using the Einstein relation to derive the minority and majority carrier mobility, and a transport model for kesterite is proposed. More details on the TRTS technique can be found in the methods section.

The proposed method works on wafers as well as thin films. The only major requirement for the samples is a relative high surface recombination. If negligible surface recombination is present a sample may be treated by etching, oxidation, or ion bombardment^6^ to induce surface recombination. In this work we lifted off a 1.55 µm thick coevaporated Cu_2_ZnSnSe_4_ film from the molybdenum coated glass substrate which leaves a bare, unpassivated, and possibly damaged surface due to the lifting process.

## Results

### The sum mobility of electrons and holes by TRTS spectra

**Figure 2:**
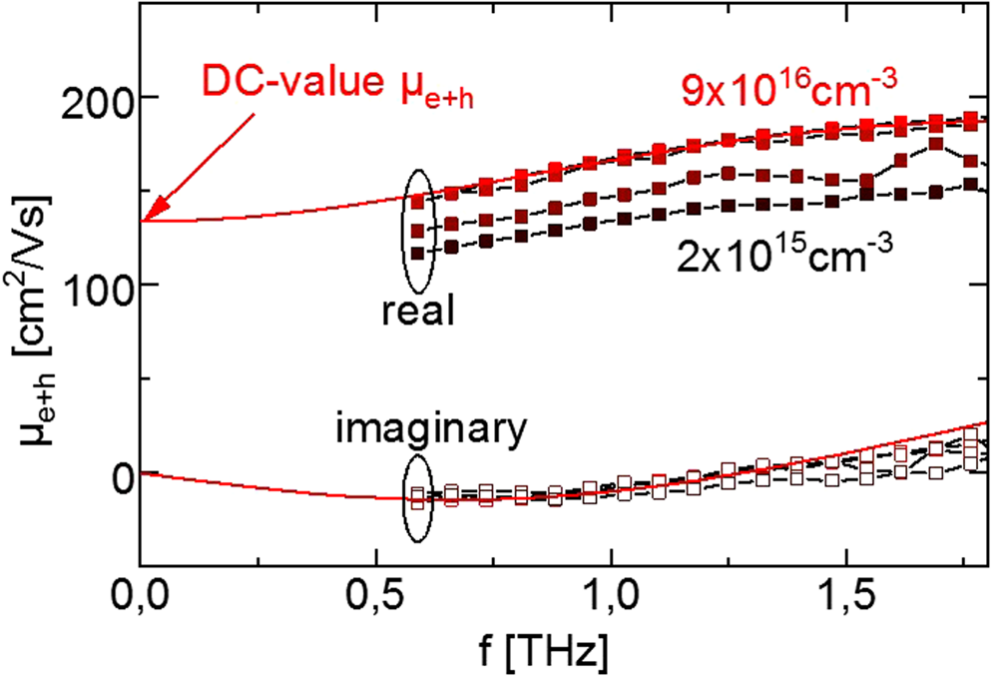
Sum of electron and hole mobility *µ*_*e*+*h*_ derived from TRTS spectra. *µ*_*e*+*h*_ at THz frequencies f on a Cu_2_ZnSnSe_4_ thin film for different injected peak carrier concentrations. The Drude-Smith model derives a DC-value of 109–135 cm^2^/Vs.

The sum of electron and hole mobility *µ*_*e*+*h*_ as function of THz frequency for the kesterite thin film is shown in Fig. 2. It contains the real and the imaginary part of the mobility. These define the AC-current of photoinduced charge carriers in response to a driving electric field at THz frequencies, as indicated in Fig. 2.

The frequency dependence of the sum mobility shows an increase with frequency for the real part and a negative imaginary part which can be assigned to partially localized charge carriers^7^. Modeling the mobility by the phenomenological Drude-Smith model for localized transport with equation (1) yields a DC-sum mobility *µ*_*DC*_ of 109–135 cm^2^/V-s with a minor injection dependence^7,8^. The slightly increased mobilities at higher injection levels may be either attributed to the saturation of localized traps as band tails and discreet defect states or to the screening of potential fluctuations which will be addressed in a future publication. Here, they contribute to the uncertainty of ±25%, which also contains the uncertainty in the initially injected carrier concentration and the uncertainty of the modeling to derive the DC-value.

1 $${\mu }_{e+h}(f)=\frac{{\mu }_{DC}}{1+{c}_{1}}\frac{1}{1+2\pi i{\tau }_{scat}\,f}(1+\frac{{c}_{1}}{1+2\pi i{\tau }_{scat}\,f})$$

Further interpretation of the localization parameter c_1_ and the characteristic scattering time τ_*scat*_ can be found in^9^, but for this work the determination of the DC-value *µ*_*DC*_ of the sum of electron and hole mobility is sufficient and we concentrate on the issue of distinguishing the electron and hole mobilities. State of the art analysis of TRTS leaves the individual contributions of electrons and holes to the mobility sum unknown. However, the distinction is essential for the application of semiconductors in optoelectronic devices. For example, charge transport in semiconductors in the dark is limited by the majority carrier mobility while transport of photoexcited charge carriers is generally limited by minority carrier mobility. Therefore, the mobility sum has to be combined with an additional transport measurement to clarify the individual mobilities of electrons and holes.

### The dominant recombination processes

The TRTS transient response offers a second way to characterize charge carrier transport if significant surface recombination occurs. In this case the transient kinetics depend on how fast excited carriers diffuse to/away from the recombination centers at the surface. A modeling approach to derive ambipolar diffusion coefficients from transient photoluminescence and time-resolved microwave conductivity has been previously shown^10^. Here, we will apply it to TRTS transients.

**Figure 3:**
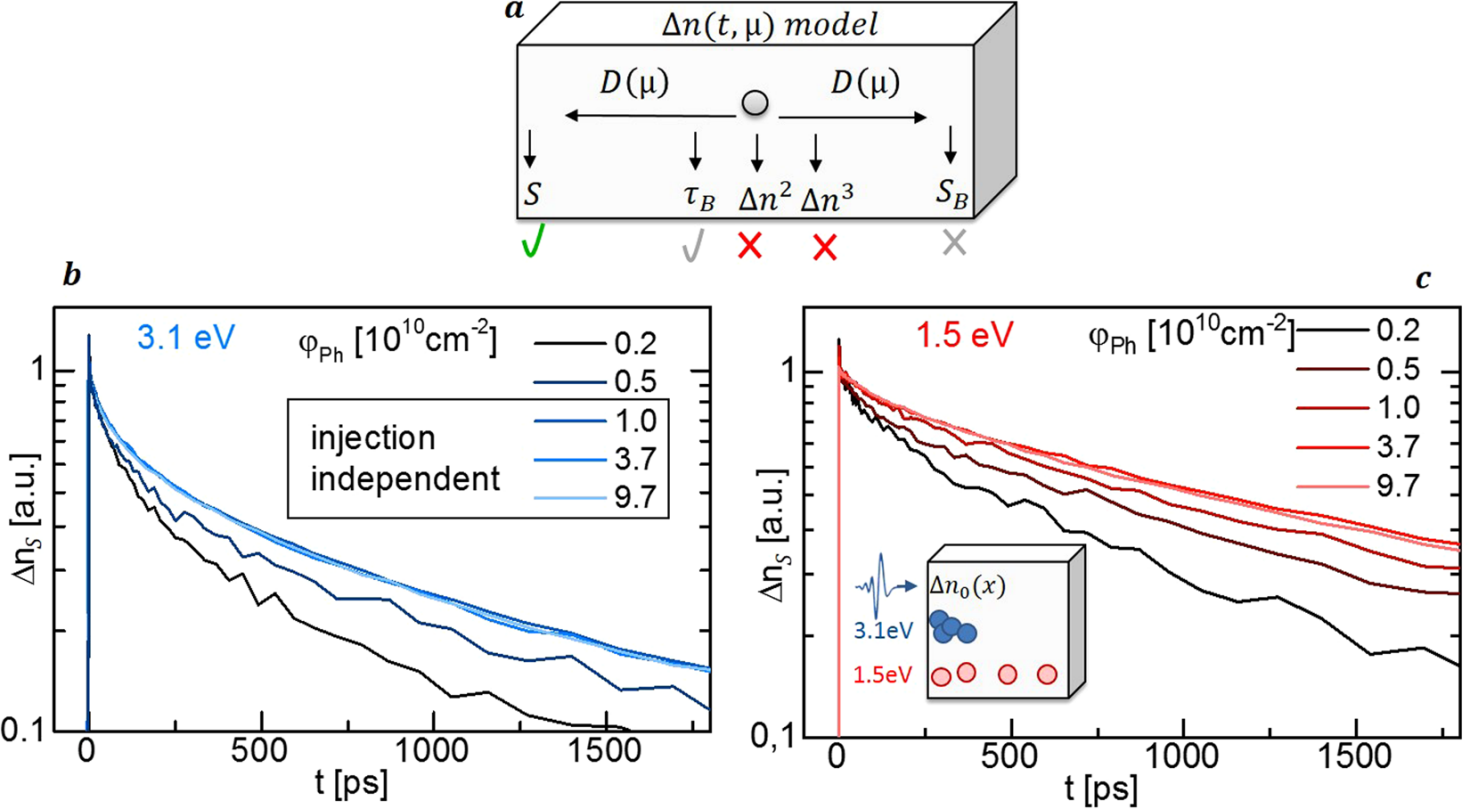
Quantitative deduction of TRTS-transient model. (**a**) Model for mobility µ dependent charge carrier transients *Δn*(*t*, *µ*) includes diffusion D and the recombination channels revealed in (**b**) and (**c**) by excitation dependent TRTS transients. Injection independent decays at high pump photon fluxes φ_Ph_ exclude higher order recombination (~Δn^2^, ~Δn^3^). Faster decays for (**b**) near-surface excitation with 3.1 eV photons than for (**c**) bulk excitation with 1.5 eV photons reveal front surface recombination S.

To verify a significant surface recombination and clarify which other recombination processes have to be included in the modeling, the TRTS transients on the kesterite film were recorded for different excitation photon energies and intensities, as shown in Fig. 3a–c. Transients are normalized at a pump-probe delay *t* of 2 ps to be able to neglect the influence of initial carrier thermalization and trapping.

TRTS transients show faster decays for 3.1 eV optical pump photons in Fig. 3b compared to 1.5 eV photons in Fig. 3c at comparable photon fluxes *φ*_*Ph*_ and excited sheet carrier concentrations *Δn*_*S*_. This behavior is explained by the shorter absorption depth for 3.1 eV photons which leads to a higher density of carriers as the absorber surface, resulting in an enhanced surface recombination rate^11,12^.

Bimolecular or Auger recombination can also be enhanced by the higher surface carrier concentration, in which case a faster decay rate would be expected for higher carrier concentrations. However, these recombination processes can be excluded by the injection dependence of the transients shown in Fig. 3, as slower transients are observed for higher photo-excited charge carrier concentrations. We attribute this behavior to injection-dependent carrier diffusion and Shockley-Read-Hall (SRH) recombination rates, discussed below, although surface band bending can result in similar behavior.

Having excluded higher order recombination processes and verified significant surface recombination, the modeling of the TRTS-transients can be limited to SRH-type surface and bulk recombination as illustrated in Fig. 3a.

Additionally, trapping into and de-trapping out of the trap states were indicated by the carrier localization and Drude-Smith like mobility observed in Fig. 2. But these effects do not have to be explicitly modeled to reproduce the transients. They can be included in the “effective bulk lifetime” *τ*_*B*_ which comprises the time the carriers live in the trap states as well as in the mobile states. Therefore, “effective bulk lifetime” may overestimate the “free carrier lifetime” in the band states as was found in a recent study on photoluminescence decay times in Cu_2_ZnSnSe_4_^13^. Such distinction between “free” and “effective” lifetime is crucial for estimating the efficiency of a kesterite solar cell.

### Direct evidence for ambipolar diffusion

Before modeling the TRTS-transients with ambipolar diffusion, it will be verified that electrons and holes diffuse together at the same speed, which is the definition of ambipolar diffusion.

**Figure 4:**
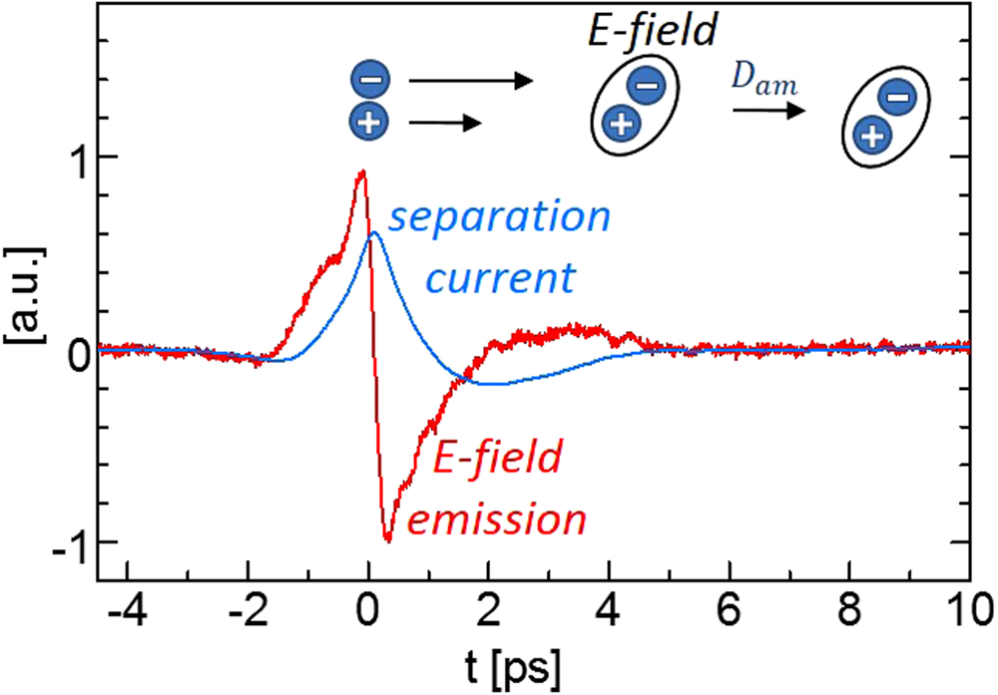
Scheme of initial ultra-fast charge transport. Excited electrons and holes diffuse apart which emits the measured E-field. The deduced (Equ.2) separation current of electrons and holes vanishes after ca. 5 ps indicating common ambipolar diffusion D_am_ of electron and holes for later times.

In general, electrons and holes diffuse at different speeds due to differences in their mobility and they have to be described by individual continuity equations coupled by the Poisson equation. However, the differing diffusion rates become self-limiting as the separation of electrons and holes results in a dipole and its electric field opposes further separation, as illustrated in Fig. 4. Subsequently, electrons and holes diffuse together with a common ambipolar diffusion coefficient *D*_*am*_, which eliminates the Poisson equation and one of the continuity equations when modeling^14^.

2 $${E}_{THz}\propto \frac{d}{dt}I=\frac{{d}^{2}}{d{t}^{2}}e(n-p)$$

Experimentally, this can be verified by THz emission spectroscopy as shown in Fig. 4. The initial diffusion of electrons and holes with different speed results in a separation current *I* and the temporal change in this current *dI/dt* causes the emission of an electromagnetic wave called photo-Dember-effect, described by equation (2)^15^. The electric field amplitude - not only the intensity - of this electromagnetic wave, broadened by the detector response function, was measured in the THz-Setup by electro-optical sampling and is shown in Fig. 4. Equation (2) allows to reconstruct the separation current which vanished after ~5 ps. It verifies that for later times no separation of electrons and holes occurs and that the diffusion is ambipolar.

### Modeling injection independent transients

Based on the results of the previous sections, we can model TRTS- transient with a joint ambipolar continuity equation for electrons and holes and surface recombination as boundary condition, as described by equations (3 and 4)^16^. These equations account for ambipolar charge carrier diffusion, bulk recombination, and surface recombination^17,18^. The charge carrier generation *g* by the pump pulse has characteristic absorption depths (1/*α*) of 230 nm and 50 nm for the 1.5 eV and 3.1 eV pump photons, respectively^19^.

3 $$\frac{d}{dt}{\rm{\Delta }}n=g-\frac{{\rm{\Delta }}n}{{\tau }_{B}}+{D}_{am}\frac{{d}^{2}}{d{x}^{2}}{\rm{\Delta }}n$$

4 $${D}_{am}\frac{d}{dx}{\rm{\Delta }}n{|}_{x=0}=-\,S{\rm{\Delta }}n$$

**Figure 5:**
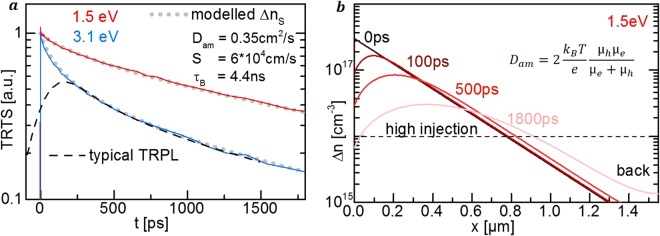
Modeling injection-independent TRTS-transients. (**a**) Injection independent TRTS transients are modeled by equations (3, 4 and 10) with a surface recombination velocity S, an ambipolar diffusion coefficient D_am_, and an effective bulk lifetime τ_B_. TRTS resolution of 0.1 ps resolves the initial decay in contrast to typical TRPL detectors with 300 ps. (**b**) Modeled charge carrier distribution *Δn*(*x*, *t*) stays in high injection and does not interact with the back surface within time span of the TRTS transients.

The last simplification is the assumption of the injection independence of the diffusion coefficient *D*_*am*_(*Δn*), the effective bulk lifetime τ_*B*_(*Δn*) and the front surface recombination velocity *S*(*Δn*). Phenomenologically, this simplification is justified when the measured TRTS transients are injection independent as it is found for the highest injection levels in our measurements shown in Fig. 3b,c. The injection independence is valid for excited carrier concentrations *Δn* above the doping *p*_0_ explained by theory in the following sections. Therefore, we will focus on the simpler injection independent modeling of a high injection pair of TRTS-transients excited with 1.5 eV and 3.1 eV photons in Fig. 5a which is sufficient to determine the electron and hole mobilities. The injection dependent modeling is shown in the Supplementary Fig. S2.

The modeling with equations (3 and 4) yields the kinetics of the charge carrier distribution *Δn*(*x*, *t*) shown in Fig. 5b. Its integration over the sample depth derives the sheet carrier concentration *Δn*_*S*_(*t*) which determines the transient TRTS signal via equation (10). The excellent agreement of the modeled and measured transients is shown in Fig. 5a,b and has a standard error of 1.2 × 10^−4^_._ This value is achieved for a surface recombination *S* of 5.9 × 10^4^ cm/s ±50%, an effective bulk lifetime or decay time *τ*_*B*_ of 4.4 ns ±15% and an ambipolar diffusion coefficient *D*_*am*_ of 0.35 cm^2^/s ±50%. These were determined by numerical minimization of the standard error. The uncertainty in *S* and *D*_*am*_ is mainly attributable to the uncertainty of the absorption coefficient at the pump wavelength which was assumed to be 10%. The uncertainty is further detailed in the Supplementary Table S1 and can be reduced by regarding three conditions.

First, the initial effective lifetime *τ*_*eff*_ approximated by equation (5)^11^ has to be dominated by the *S* and *D*_*am*_ terms to assure significant impact of surface recombination and diffusion. These terms correspond to the time *τ*_*DF*_ to diffuse to the front surface and *τ*_*S*_ to recombine at the front surface.

Second, the use of a pair of transients excited at different absorption coefficients α_1_ ≪ α_2_ reduces the interdependency of the estimated *D*_*am*_ and *S* values.

Third, estimating τ_*B*_, *D*_*am*_ and *S* by numerical modeling of the transients is less uncertain than by using the approximation (5) for the effective lifetime at initial times when carriers are distributed over α^−1^ and in the long time limit t > *τ*_*DB*_ when carriers have diffused over the sample thickness *d* to the back surface. Later ones may be taken as start value for the numerical modeling.

5 $$\frac{1}{{\tau }_{eff}}\approx \frac{1}{{\tau }_{B}}+\frac{1}{{\tau }_{S}+{\tau }_{DF}}=\frac{1}{{\tau }_{B}}+\frac{1}{\frac{1}{2S\alpha }+\frac{1}{{\pi }^{2}{D}_{am}{\alpha }^{2}}}with\,{\alpha }^{-1}\to d\,for\,t\to {\tau }_{DB}=\frac{{d}^{2}}{{\pi }^{2}D}$$

For consistency it was confirmed in Fig. 5b that the majority of the carriers stays in high injection throughout the whole modeling period. If not, the modeling can be limited to an initial time span and the transients at later times can be disregarded to avoid a decay into the injection dependent regime.

This procedure also excludes back surface recombination if the carriers do not have the time to diffuse to the back. In the presented modeling the 1.8 ns time window is much shorter than the diffusion time to the back surface *τ*_*DB*_ of 7 ns and the carrier distribution at 1.8 ns has not reached the back as shown in Fig. 5b. Also, grain boundaries are not crossed for the majority of charge carriers in the 1.55 µm thick kesterite thin film with an approximate grain size of 1 µm. Hence, the derived diffusion coefficient corresponds to vertical intra-grain diffusion.

In principle, similar analyses can be performed on time resolved photoluminescence (TRPL) transients. In^20^ a surface recombination velocity of 2 × 10^4^ cm/s was derived from TRPL transients of similar kesterite samples based on the analytic approximation (5) of the continuity equation^12,18,21^. However, the time resolution of a TRPL detector can lead to an underestimation of the initial decay and the TRPL maximum can occur significantly after carrier excitation, as shown in Fig. 5a, by convoluting the TRTS transient with typical TRPL response of ca. 300 ps full width half maximum. Both effects oppose a reliable mobility analysis and show the benefits of superior time resolution of TRTS.

### Minority and Majority Carrier Mobility by ambipolar Einstein Relation

Now the individual carrier mobilities will be derived from the ambipolar diffusion coefficient and the mobility sum.

**Figure 6:**
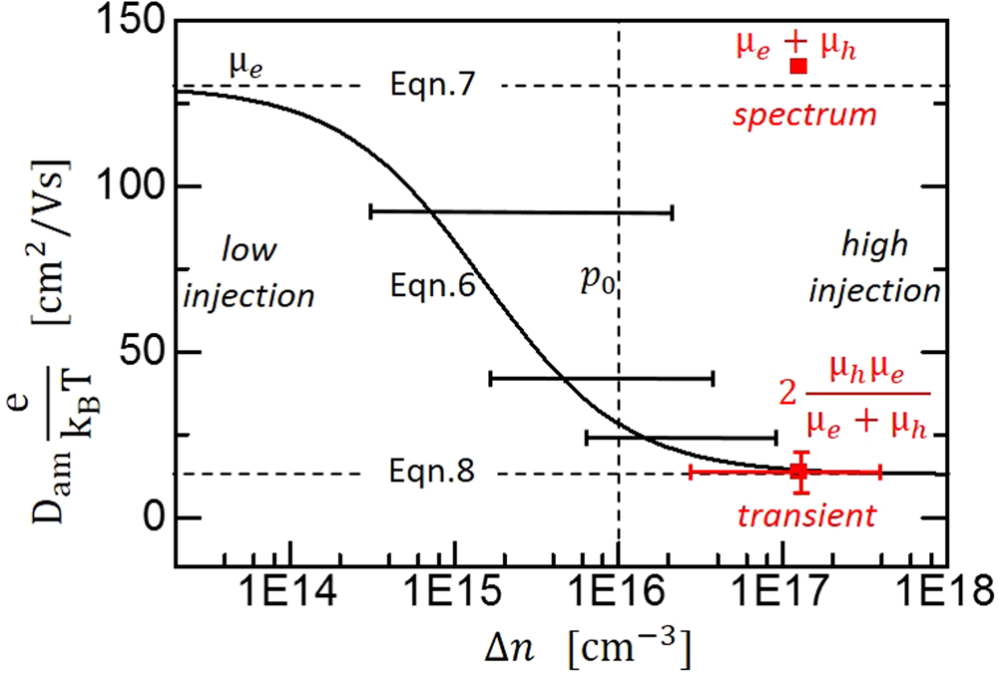
Combining TRTS spectra and transients in high injection. Injection dependence of the ambipolar diffusion coefficient D_am_ given by equation (6) and its limits equations (7 and 8) in low/high injection. D_am_ derived from TRTS transients (red square) and the mobility sum derived from the TRTS spectrum can be combined with equation (8) to yield the mobility for electrons µ_e_ and holes µ_h_. Horizontal bars correspond to the decay of the peak carrier concentration within the 1.8 ns TRTS transients and visualize the large uncertainty of the diffusion coefficients derived in the injection-dependent regime.

The contributions of electron *µ*_*e*_ and hole *µ*_*h*_ mobility to the common ambipolar diffusion coefficient *D*_*am*_(*Δn*) are dependent on the injection *Δn*. They are given by the ambipolar Einstein equation (6) for p-type semiconductors. The injection-dependence of *D*_*am*_(*Δn*) following equation (6) is shown in Fig. 6 by the black curve^14,22^. For n-type semiconductors, the n and p labels interchange in equation (6).

6 $${D}_{am}({\rm{\Delta }}n)=\frac{{k}_{B}T}{e}\frac{2{\rm{\Delta }}n+{p}_{0}}{\frac{{\rm{\Delta }}n}{{\mu }_{p}}+\frac{{\rm{\Delta }}n+{p}_{0}}{{\mu }_{n}}}$$

However, in the limits of relative high and low injection levels *Δn* the contributions of the electron *µ*_*e*_ and hole *µ*_*h*_ mobility to *D*_*am*_ become independent of the injection *Δn* and the in generally unknown doping concentration *p*_0_. The injection independent modeling of TRTS transients in relatively high injection in the previous section has already taken advantage of this behavior.

For low injection, *Δn* ≪ *p*_0_*µ*_*p*_*/µ*_*n*_, *D*_*am*_(*Δn*) reduces to equation (7) and the diffusion coefficient is dominated by the minority carrier mobility. For high injection *Δn* ≫ *p*_*0*,_ *D*_*am*_(*Δn*) reduces to equation (8) and is dominated by the mobility of the carrier type with the smaller mobility, as shown in Fig. 6.

7 $${D}_{am}={\mu }_{e}\frac{{k}_{B}T}{e}\,for\,{\rm{\Delta }}n\ll \frac{{\mu }_{h}}{{\mu }_{e}}{p}_{0}$$

8 $${D}_{am}=2\frac{{\mu }_{h}{\mu }_{e}}{{\mu }_{e}+{\mu }_{h}}\frac{{k}_{B}T}{e}\,for\,{\rm{\Delta }}n\gg {p}_{0}$$

The diffusion coefficient of 0.35 cm^2^/s derived by modeling TRTS transients in high injection conditions (Fig. 5) can be inserted in equation (8) and combined with the sum mobility of *µ*_*e*_ + *µ*_*h*_ = 135 *cm*^2^*/V-s* derived by TRTS spectra thus yielding mobility values of 7.3 cm^2^/V-s ±50% and 128 cm^2^/V-s ±25%.

However, equation (8) does not determine which of these two values corresponds to electrons and which to holes. To distinguish them, we have also modeled pairs of TRTS-transients in the injection-dependent regime with an effective injection-independent *D*_*am*_, which results in the black bars in Fig. 6. This modeling is only a rough estimate as the *D*_*am*_ changes from the initial to the final peak carrier concentration after 1.8 ns which is indicated by the error bars in Fig. 6. However, the trend is clear: it shows that the diffusion coefficient increases towards the lower injection level where the diffusion is dominated by the minority carriers. Therefore, we conclude that electrons have the larger mobility of 128 cm^2^/V-s and holes the smaller mobility of 7 cm^2^/V-s.

A similar analysis works for low injection transients where the effective mobility can directly be identified as the minority carrier mobility in equation (7). However, high injection has the advantages of larger signals in the TRTS measurement, screened surface fields, and the condition *Δn* ≫ *p*_0_ does not depend on the (unknown) mobilities as does the condition for low injection *Δn* ≪ *p*_0_*µ*_*p*_*/µ*_*n*_.

The injection dependent transients can also be analyzed to estimate the doping concentration *p*_0_. Due to the limited space in this article it is given in the Supplementary Fig. S2.

### Interpretation of the TRTS-derived mobilities

To interpret the TRTS-derived mobilities in polycrystalline thin films the distinction of vertical vs. lateral and intra-grain vs. cross grain boundary transport is important as we have discussed in^9^.

**Figure 7:**
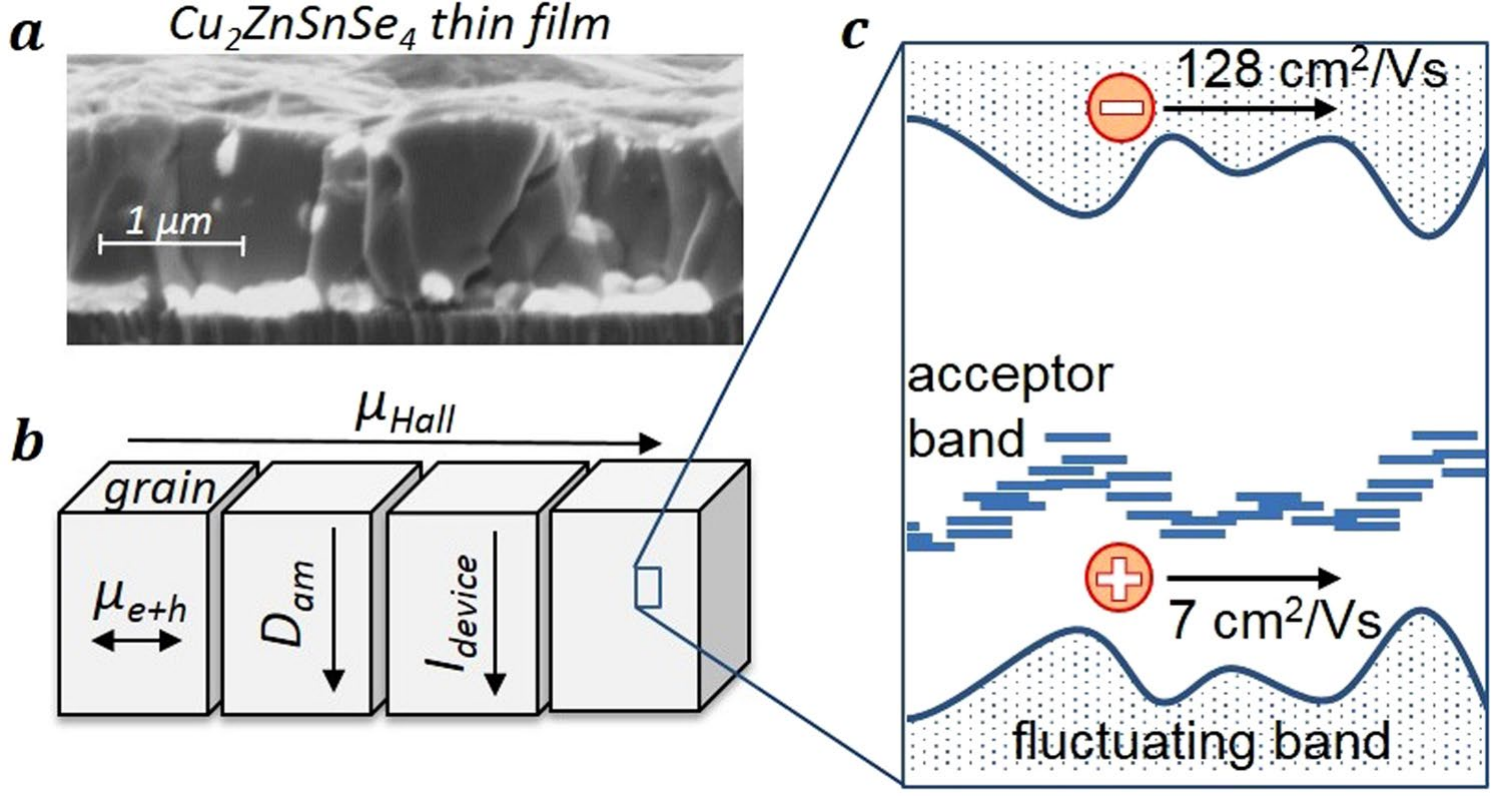
Transport model for Cu_2_ZnSnSe_4_. (**a**) SEM picture of the polycrystalline kesterite thin film. (**b**) TRTS derived mobilities describe intra-grain transport as usual for many commercial devices. Hall derived mobility describe lateral transport across grain boundaries. (**c**) Model for intra-grain transport in kesterite with partially localized but relatively fast electron transport in a fluctuating conduction band and slower hole transport in an acceptor band.

The added mobility of electrons and holes derived by the terahertz absorption spectrum in Fig. 2 is based on lateral acceleration of charge carrier on the nm-scale. Hence, it yields the intra-grain mobility for the µm-sized grains in the polycrystalline thin film shown in Fig. 7a,b.

The ambipolar mobility derived by the terahertz transients is based on vertical ambipolar diffusion into the thin film. As discussed above, the charge carriers do not cross grain boundaries within the time window of the TRTS measurement, allowing to identify the derived mobilities with the intra-grain mobilities, too^23^.

Hence, the combination of both measurements yields isotropic intra-grain mobilities of electrons as well as of holes.

These are relevant mobilities and transport distances for most commercial applications of polycrystalline thin films such as photovoltaics, solar water splitting devices or LEDs. Their charge current *I*_*device*_ in operation is vertical and occurs within single grains if these extend throughout the thin film thickness as shown for our sample in Fig. 7a,b.

Minority carrier mobilities for vertical transport may also be estimated from a combination of capacitance-voltage, internal quantum efficiency and time resolved photoluminescence measurements on completed solar cells^24^. Electron mobilities of ca. 100 cm^2^/Vs have been derived from this method for similar kesterite thin film samples^25^ comparing well to the TRTS-derived value of 128 cm^2^/Vs and thus underlining the validity of the current approach.

In contrast, Hall effect measurements — as the most common method to derive majority carrier mobility — involve lateral transport which in polycrystalline thin film materials may be dominated by thousands of grain boundaries illustrated in Fig. 7b. As stated above, this is not the relevant transport direction for photovoltaic thin film devices. The huge variation from 0.5 to 104 cm^2^/Vs^5,26–30^ in the reported majority carrier Hall-mobility in kesterites may therefore be caused by either a variation of the intra-grain transport or by grain boundary barriers depending on the growth conditions.

The absolute values of the TRTS-derived intra-grain mobilities of electrons and holes are significantly smaller than what is expected from classical (Drude) free carrier transport, were the mobility is directly linked to the effective mass and carrier scattering time. The non-Drude behavior is already obvious from the THz spectra in Fig. 2, where the negative imaginary part indicates localization of carriers. This was previously explained in^9^ by the influence of potential fluctuations as illustrated in Fig. 7c.

Additionally, the TRTS-derived hole mobility is 20 times smaller than the electron mobility, although the theoretical effective mass is only 2 times smaller. This behavior can be explained by much stronger fluctuations of the valence band compared to the conduction band. Alternatively, the hole transport may be dominated by transport in an acceptor band which has been recently identified to be present in the related material Cu_2_ZnSnS_4_^31^. We note that a similar hole mobility of 5 cm^2^/Vs has been reported for transport in an acceptor band of strongly Mg doped GaAs^32^.

In summary, we have developed a contact-less method to measure both minority (7 cm^2^/V-s ±50%) and majority (128 cm^2^/V-s ±25%) charge carrier mobility and demonstrated it for an example of a p-type Cu_2_ZnSnSe_4_ thin film. The results of this method agree well with electron mobilities derived from carrier collection in Cu_2_ZnSnSe_4_ solar cells. The method gives access to the intra-grain and vertical transport in a polycrystalline thin film. The MatLab code for the underlying TRTS transient modeling is freely available on request to the corresponding author and can be applied to any semiconductors with sufficient or, as in this case, induced front surface recombination. This modeling technique additionally determines the surface recombination velocity (5.9 * 10^4^ cm/s), the effective bulk lifetime (4.4 ns) and estimates the doping (ca. 10^16^ cm^−3^). Therefore, TRTS is an excellent technique to reveal charge carrier dynamics. In our example, we could explain the partially localized electron transport and the 20 times smaller hole mobility revealed in Cu_2_ZnSnSe_4_ by band edge fluctuations and an acceptor band.

## Method

Our TRTS setup uses an 800 nm or 400 nm pump pulse, a THz probe pulse and an 800 nm sampling pulse. The optical pump pulse photo-excites electrons and holes in the sample. The THz pulse probes the mobility *µ* and concentration *Δn* of the excited charge carriers via a change *ΔT* in the THz transmission *T*, or alternatively a change in its reflection. The third pulse samples the electric filed of the THz pulse by electro-optical sampling in a ZnTe crystal to detect the THz pulse and its transmission through the sample.

Scanning the delay time *t* between THz probe pulse and optical pump pulse by a delay line yields the TRTS-transients.

The delay *t*_2_ between THz and sampling pulse defines the sampled part of the THz pulse (e.g. the maximum). Scanning this second delay reveals the whole THz pulse transmitted through the sample and therefore the transmission *T*(*t*_2_) and *ΔT*(*t*_2_) in time domain which is then Fourier transformed to *T*(*f*) and *ΔT*(*f*).

Due to the long wavelengths of the THz radiation (1 THz ↔ 0.3 mm), *ΔT*(*f*, *t*) is dominated by free carrier absorption which is proportional to the photo-induced conductivity *Δσ* = *eµΔn* consisting of the charge carrier mobility *µ* and concentration *Δn*^33^. Therefore, the measured THz transmission *T* and its photoinduced change *ΔT* can be used in equation (9) to calculate the photo-induced conductivity as well as the mobility if the induced charge carrier concentration is known^33^.

9 $${\rm{\Delta }}{\sigma }_{S}(t,f)=e\ast {\mu }_{e+h}(f,t)\ast {\rm{\Delta }}{n}_{S}(t)={\varepsilon }_{0}c({n}_{0}+{n}_{2})\frac{{\rm{\Delta }}T(t,f)}{T+{\rm{\Delta }}T}$$

Equation (9) is often referred to as the thin film approximation. It depends on the speed of light *c*, the vacuum permittivity *ε*_0_ and the THz refractive indices of the surrounding medium *n*_0_ and of the substrate of the excited film *n*_2_. It is prevalent to state equation (9) with a carrier concentration *Δn* and conductivity *Δσ* assumed to be homogenously distributed over the layer thickness *d* (or alternatively over the absorption depth *1/α*). However, within the thin film approximation the specific distribution doesn’t affect the THz absorption. Hence, only the integrals of *Δσ* and *Δn* over depth are of relevance. Therefore we state equation (9) with the sheet charge carrier concentration *Δn*_*S*_(*t*) and the integral conductivity *Δσ*_S_.

10 $${\rm{\Delta }}{n}_{S}={\int }_{0}^{d}{\rm{\Delta }}n(x)dx\,={\varphi }_{ph}(1-r-t)$$

To derive the mobility from equation (9) the THz transmission *T and ΔT*(*t* ≈ *0*) is measured shortly after excitation (20 ps) and the initial sheet carrier concentration *Δn*_*∫*_(*t* = *0*) is calculated by equation (10) with the flux of the pump photons *φ*_*ph*_ reduced by their transmission t and reflection *r* at the sample. The transients were calibrated as shown in the Supplementary Fig. S1.

## Electronic supplementary material

Supplementary Information

## Data Availability

The datasets generated and analyzed during the current study are available from the corresponding author.
