# Supplementary material for: Minority and Majority Charge Carrier Mobility in Cu2ZnSnSe4 revealed by Terahertz Spectroscopy
Source: Sci Rep. 2018 Sep 27;8:14476. doi: 10.1038/s41598-018-32695-6 (PMC6160416; doi:10.1038/s41598-018-32695-6)
Supplement: Supplementary file 1 — Supplementary Information [file 41598_2018_32695_MOESM1_ESM.pdf]

Supplementary Information

Minority and Majority Charge Carrier Mobility in $Cu_2ZnSnSe_4$ revealed by Terahertz Spectroscopy

Hannes Hempel[1], Charles J. Hages[1], Rainer Eichberger[2], Ingrid Repins[3], Thomas Unold[1]**

[1] Department Structure and Dynamics of Energy Materials, Helmholtz-Zentrum Berlin für Materialien und Energie GmbH, Hahn-Meitner-Platz 1, 14109, Berlin, Germany
[2] Institute for Solar Fuels, Helmholtz-Zentrum Berlin für Materialien und Energie GmbH, Hahn-Meitner-Platz 1, 14109 Berlin, Germany
[3] National Renewable Energy Laboratory, 15013 Denver West Parkway, Golden, CO 80401-3305, U.S.A.
**hannes.hempel@helmholtz-berlin.de *unold@helmholtz-berlin.de*

Calibration of TRTS transients

The presented method relies on the accurate measurement of the transients and TRTS uses a mechanical delay of the pump beam to realize time resolution. However, this mechanical delay can result in a change of pump spot size or the overlap of THz probe and optical pump pulse which obscures the original transient. To account for these effects we calibrated TRTS-transient on a Si wafer sample with a µs lifetime. Fig. S1 shows the TRTS-transient of the silicon wafer reference, the quadratic polynomial fit to the reference transient, the transient as measured on the kesterite sample, and the sample transient calibrated by the fit of the silicon reference.

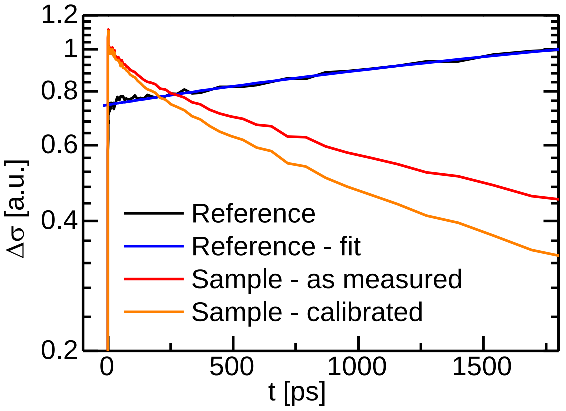

Fig. S1: Calibration of the TRTS-transients.

Uncertainty estimation

The uncertainties in the modelled properties D_{am}, S and T_b are estimated from the uncertainties of ±10% in the input parameter which are the absorption coefficient at 400 nm α_{400nm} and 800 nm α_{800nm} and the thin film thickness d. Table S1 shows that a change of ±10 % in the absorption coefficients causes a relative large change in the derived D_{am} and S of up to 38 %. The combined uncertainties of S and D_{am} are ca. ±50 %.

The uncertainty in the hole mobility μ_h is dominated by the uncertainty in D_{am} of ±50 %. The uncertainty in the election mobility μ_e is dominated from the uncertainty in the sum mobility μ_{e+h} of ca. 25 %.

input parameter		modelled properties		
		D_{am}<br>0.35cm/s	S<br>5.9x10^{4}cm/s	T_b<br>4.6 ns
α_{800nm}	+10%	-30%	-30%	-2%
4.4x10^{4}cm^{-1}	-10%	+38%	+38%	+4%

α_{400nm}	+10%	+10%	+10%	-6%
$2x10^5 cm^{-1}$	-10%	-19%	-19%	+10%
d	+10%	-1%	-2%	-5%
1.55µm	-10%	-1%	-2%	-5%

Supplementary Tab. S1: Uncertainty of the properties derived by the TRTS-transient modelling.

Estimate of doping from injection dependent transients

Modelling injection dependent transients at intermediate injection levels $\Delta n \approx p_0$ can in principle yield the doping concentration p_0 which is a key property of semiconductors, and its contactless derivation is very desirable.

However, modelling the injection dependence is complex and requires combining the continuity equations (3-4) with the injection dependence of the diffusion coefficient $D_{am}(\Delta n)$ in equation (5). Additionally, also the effective bulk lifetime $\tau_B(\Delta n)$ in equation (S1) and the surface recombination velocity $S(\Delta n)$ in equation (S2) depend on the local carrier concentration.

Based on Shockley-Read-Hall recombination at a discrete defect level in the bulk and at the surface the effective bulk lifetime $\tau_B(\Delta n)$ is given by the capture times of τ_p of holes and τ_e of electrons and the parameter p_t in equation (S1) connected to reemission of charge carriers from the defect level [1]. A similar equation (S2) relates the effective surface recombination velocity and the surface recombination velocities of electrons and holes.

$$\tau_B(\Delta n) = \tau_h \frac{\Delta n}{p_0 + p_t + \Delta n} + \tau_e \qquad (S1)$$

$$\frac{1}{S(\Delta n)} = \frac{1}{S_h} \frac{\Delta n}{p_0 + p_t{}' + \Delta n} + \frac{1}{S_e} \qquad (S2)$$

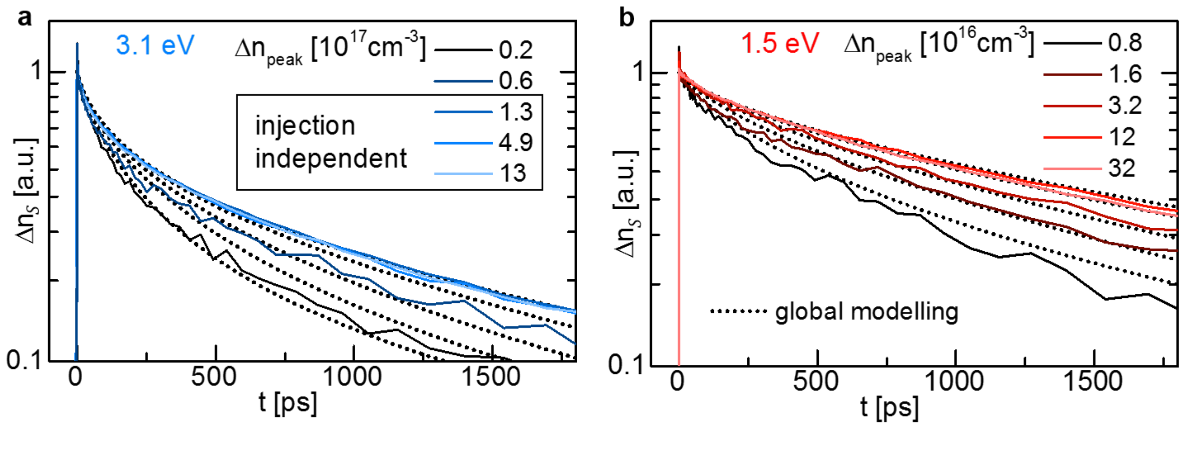

Fig. S2: Global injection-dependent modelling of TRTS-transients. *Same injection dependent TRTS-transients as in Fig.3 now shown with a common global modelling (dotted line) for a) and b) based on equations (3,4,5,S1,S2) and labelled with the initially excited peak carrier concentration at the sample surface $\Delta n_{peak.}$ The TRTS-transients start to differ from the high injection behaviour when they have decayed to ca. $10^{16} cm^{-3}$ which is an estimate for the doping concentration.*

A qualitative agreement to the measured TRTS transients is found for $p_0 = 10^{16}\, cm^{-3}$, $S_h = 7\times10^4\, cm/s$, $S_e = 3\times10^5\, cm/s$, $\tau_e = 5\, ns$, $\mu_e = 129\, cm^2/Vs$, $\mu_h = 6\, cm^2/Vs$ and $p_t = p_t{}' = \tau_h = 0$ as shown by the dotted lines in Fig.S2. However, due the large amount of 9 parameters and the limited computation power we cannot exclude that another parameter combination reproduces the measured transients with a smaller the standard error than this parameter set. Additionally a more advanced model should include

a distribution of tail states instead of a single discrete defect level and also trapping effects which have been shown to increases the effective bulk lifetime τ_B particularly in kesterites [2]. On the flipside, these more advanced models require even more parameters and modelling may become unambiguous.

Still the general trend of the injection dependence is clear and exhibits the transition from injection-independent transients in high injection to the injections dependence at $\Delta n \approx p_0 \approx 10^{16}\,cm^{-3}$. These general trend described by equations (5,S1,S2) is caused by the transition from the dominance of the minority carriers in low injection to the impact of both carrier types in high injection. In the case of the p-type kesterite sample these dynamics are given by $D_{am} \sim \mu_e$, $S = S_e$ and $\tau_B = \tau_e$ in low injection and $D_{am} \sim 2\mu_h$, $S^{-1} = S_e^{-1} + S_h^{-1}$ and $\tau_B = \tau_e + \tau_h$ in high injection. For the estimation of p_0 it is not relevant if the change in the transients is due to a change in diffusion, effective bulk life time or surface recombination velocity. It is only relevant that all these changes take place at $\Delta n \approx p_0 \approx 10^{16}\,cm^{-3}$. Therefore, this value maybe taken as a rough estimate of the doping and is in line with the value derived from capacitance-voltage measurements on the completed solar cell.

References

[1] Shockley, W. & Read Jr, W. T. Statistics of the recombinations of holes and electrons. Phys. Rev. **87,** 835 (1952).

[2] Hages, C. J. et al. Identifying the Real Minority Carrier Lifetime in Nonideal Semiconductors: A Case Study of Kesterite Materials. Adv. Energy Mater. 1700167 (2017).
